# Choice for the horse is paramount: Owners’ experiences in facilitating friends, forage, and freedom in their management practices

**DOI:** 10.1017/awf.2026.10107

**Published:** 2026-08-10

**Authors:** Wendy Leah Watson, Lesley Jane Jessiman, Cathy Dwyer

**Affiliations:** 1 Royal Dick School of Veterinary Studies/Roslin Institue, The University of Edinburgh Royal Dick School of Veterinary Studies, United Kingdom; 2 SRUC, Animal Behaviour and Welfare School of Veterinary Medicine and Biosciences King’s Buildings West Mains Road Edinburgh, UK EH9 3JG, Scotland’s Rural College, United Kingdom

**Keywords:** Animal welfare, anthropomorphism, horse husbandry, human-horse relationship, interviews, livery yard culture

## Abstract

Owned horses rely on people for their behavioural needs, such as access to conspecifics, constant access to low nutrient density, fibrous forage and freedom of movement; otherwise known as friends, forage and freedom (3Fs). Restricting the 3Fs prevents horses from achieving optimal welfare. We carried out 14 semi-structured interviews in the UK and Ireland to examine horse owners’ experiences, attitudes and beliefs around the provision of the 3Fs. Most participants were female, aged from 25 to 65 years, resident in the UK and kept their horse at a livery facility. A reflexive thematic analysis identified five core themes in their responses. Themes identified were ‘Choice for the horse is paramount’, ‘Friends, forage, freedom and fear of injury’, ‘When livery yard rules compromise horse welfare’ (sub-themes ‘Control of forage and freedom of movement’), ‘Horse management through the (anthropomorphic) human lens’ and ‘The livery yard – balancing both horse and human needs’. Participants appeared to have a good understanding of the horse’s behavioural needs and were keen to communicate that these needs should be a primary consideration in their management practices. Owners who kept their horses at home stated that their horses should have access to freedom of movement and shelter in their environment. Livery yard practices, a concern for injury in group turn-out, anthropocentric, anthropomorphic and motivational beliefs all appeared to play a role in owners’ decisions for providing the 3Fs. These findings highlight the difficulties horse owners experience when wishing to implement best practice management and provide a better understanding of how such welfare challenges can be addressed.

## Introduction

In contemporary times, horses are largely used for recreation, sport, work, production or companionship (Collins *et al.* 2008; Rioja Lang *et al.* 2020). In the UK and Ireland, recreational horses are thought to make up approximately two-thirds (631,000) and one-third (33,169) of the equine population, respectively (Corbally & Fahey 2017; British Equestrian 2023). Research on the human-horse relationship often focuses on what horses can do for people in the form of providing recreation and companionship, both physical and psychological (Burgon 2003; Kendall *et al*. 2014). However, there are fewer studies which highlight people’s knowledge, understanding and implementation of the horse’s behavioural needs and welfare (Hemsworth *et al*. 2015; Ross *et al.* 2023).

Horses have evolved as social, free-roaming herbivores, who will travel approximately 16–18 km per day seeking forage and water sources (Boyd & Keiper 2005). Domestication of the horse has not changed the animal’s basic behavioural needs (Goodwin 1999), and, given the right set of circumstances, horses will maintain such behaviours in a domestic environment (Waran 1997). Domestic management practices that do not prioritise the horse’s behavioural needs can cause suffering and compromised welfare in the horse (McGreevy *et al*. 1995; Goodwin *et al*. 2002; Krueger *et al.* 2021). Restricted access to conspecifics, reduced provision of a low nutrient density/fibrous forage source during a 24-h period, and confinement to individual stabling for extended periods of time, have all been identified as resulting in poor welfare for horses (Goodwin *et al*. 2002; Krueger *et al.* 2021; Ermers *et al.* 2023). Meeting the welfare needs of the managed horse is primarily in the hands of the horse owner or manager, who may or may not have an adequate understanding of these needs, have the husbandry knowledge to ensure good welfare or the motivation to do so (Hausberger *et al.* 2015; Horseman *et al.* 2016; Luke *et al.* 2024).

The welfare consequences surrounding the restrictions of the horse’s physiological and behavioural needs have been well documented, e.g. basic horse behavioural needs (Krueger *et al.* 2021), forage requirements (Ermers *et al.* 2023; Verwijs 2024), and the friends, forage and freedom (3Fs) (Phelipon *et al.* 2024). However, much less is understood about the reasons behind horse management choices made by owners (Ross *et al.* 2023). Poor welfare decisions have been shown to result from owner/manager inexperience, lack of knowledge, attitudes, beliefs and/or owner convenience (Goodwin 1999; Visser & Van Wijke 2012; Leme *et al.* 2014; Hockenhull & Creighton 2015; Rioja Lang *et al.* 2020; Hemsworth *et al.* 2021; Mazzola *et al.* 2021; Ross *et al.* 2023). Poorer welfare choices for horses may also occur as a result of human needs taking priority over the animals’ needs. Luke *et al.* (2023) suggested that motivational priorities, such as competitiveness and control, create a disparity between the horse’s needs vs the human’s needs. Mauricio *et al.* (2024) suggested that although equine practitioners and enthusiasts (i.e. horse owners, riders or trainers) appeared to have a good understanding of horse welfare and best practice management, they often failed to translate that knowledge into practice. Key factors, such as a lack of qualified staff caring for horses in facilities, insufficient time, lack of financial resources, and differences in attitudes around the monetary value of a horse, appeared to have contributed to horse welfare issues in their study (Mauricio *et al.* 2024). In other studies, although horse owners appear to recognise the benefits of group housing for improved horse welfare, most still chose to keep horses in individual stabling (Visser & Van Wijke Janssen 2012). Ross *et al.* (2023) found that horse owners conceded that outdoor group housing was beneficial for horse welfare but were also were less convinced that outdoor group housing offered a better standard of care than individual indoor housing. Various studies suggest that even though individuals may know the better welfare options for horses they can lack the ability to follow through with this knowledge; which may be due many different reasons, such as tradition and habits (Watney *et al.* 2025), cognitive dissonance (Cheung *et al.* 2025), communication preferences (Pickering & Hockenhull 2020), and resource constraints (Mauricio *et al*. 2024; Hogg *et al*. 2025).

In England and Wales, a recent report by the Royal Society for the Prevention of Cruelty to Animals (RSPCA; 2025) suggested one of the primary equine welfare concerns was a lack of livery facilities that could provide substantial access to grazing, socialisation with other horses and freedom of movement. In our recent study (Watson *et al.* 2026) we also found that there were smaller numbers of horse owners who were providing less than 6 h of turn-out for their horses, with little access to a forage source and limited or no social interaction with other horses, which suggests a welfare concern. Similarly, other studies also suggest comparative results; horses not receiving adequate opportunity for turn-out, access to conspecifics or the right type/amount of forage (Visser & Van Wijke 2012; Leme *et al.* 2014; Hockenhull & Creighton 2015; Hemsworth *et al.* 2021; Mazzola *et al.* 2021; Ross *et al.* 2023; Mauricio *et al.* 2024; RSPCA 2025).

Therefore, the aim of this present study is to explore UK and Irish horse owners’ experiences, opinions and beliefs around their management practices, with a specific focus on their provision of the 3Fs.

## Materials and methods

### Ethical considerations

A favourable ethical opinion was obtained by Human Ethical Review Committee (HERC) from the Royal Dick School of Veterinary Studies R(D)SVS - HERC_2022_77.

Participants provided informed consent prior to data collection, were notified that they could withdraw at any time, were assured that their data would be stored securely and that the data were fully anonymised to prevent any subsequent identification.

### Design

This study was an exploratory, qualitative study using Reflexive Thematic Analysis (RTA), in which we adopted a deductive and inductive approach (Braun & Clarke 2019).

### Participants

Participants were recruited using convenience sampling via online horse forums. Participant exclusions included any SRUC (Scotland’s Rural College) or R(D)SVS staff, to ensure no previous knowledge of the current study. Participants under the age of 18 years (i.e. minimum age of consent), those residing outside the UK or Ireland, or those who did not own/manage one horse or pony were also excluded.

### Procedures

A total of fourteen semi-structured interviews were conducted by the first author (WW), from September 2022 to early February 2023, to determine the challenges owners face in their horse management practices. WW circulated an online poster constructed on Jisc, Bristol, (UK, v2) on social media platforms, such as horse Facebook groups, Twitter (*circa* 2022) and online horse forums, such as *Horse and Hound*, during June, July and August of 2022. The poster contained a link to Jisc online surveys which provided further participant information, consent and requested data on gender, location in the UK or Ireland, if participants managed a horse or pony, whether they kept their horse at home or at livery, and if the participants were interested in being interviewed, provision of their email address for contact (see Supplementary material). There was a total of 31 responses to the online survey and after one exclusion, a respondent under the age of 18 years, WW contacted the other 30 respondents via email, Microsoft Outlook®, with a request to interview. If one respondent did not reply after 3 days, WW contacted another respondent. At the end of this procedure, a total of 14 participants agreed to be interviewed for the study.

To ensure anonymity, participants were assigned a number between 1 and 14 for the analysis. Participants who kept their horses at home had an (H) beside their participant number and those who kept their horses at livery, had an (L) beside their participant number. Eleven semi-structured interviews were recorded in Microsoft Teams® (version 4.2.4.0), one interview was conducted via Skype, and two participants did not wish to be recorded online. Therefore, one interview was conducted using questions via email on Microsoft Outlook® and the other interview was conducted, but not recorded, on WW’s iPhone Plus 6, with handwritten notes taken during the conversation. The interview conducted via email involved the participant providing written answers to the questions, and was conducted in a time-frame between September 27th and October 5th, 2022. Handwritten notes were subsequently typed onto Microsoft Word®, and all recordings were added to Otter.ai (2023) for transcribing. All the transcriptions were then transferred to NVivo Version 11 (Version 11 Lumivero 2015) for coding and constructing the themes.

At the beginning of each interview and after an initial introduction, in which the interviewer got to know the participant through some informal conversation, the interview commenced (see Table 1).

**Table 1.** Interview schedule which lists questions asked of horse owner participants (n = 14) in semi-structured interviews investigating horse management challenges conducted September 2022–February 2023. Table 1. long description.

Interview schedule
Ice breaking conversation, a welcome, thank you for joining me for the interview. * How’s the weather where you are today?
How many horses do you own/manage? *Number of horses, age breed, sex. *How long have you owned these/this horse(s)? *If manage more than one horse, are they managed in the same way, or differently? *Why or why not?
Tell me what you like to do with your horses? *Competition – what type – dressage, show jumping? Primarily leisure or games, unaffiliated competition or hacking? *Why do you like to do these things with your horse? *Does this affect the way your horse is managed?
How are your horses managed? *Do you have control of this or does someone else? *Who and reasons why *Are you horses turned out daily with other horses – why or why not? * How many hours would your horse have access to turn-out and where would that be – pasture, paddock? *What type of forage source is your horse fed i.e., grass, hay, haylage, anything else? *Would you like to see your horse have more access to friends, forage and freedom? *What is preventing this?
Tell me about what the biggest challenges are in managing your horse the way you would like to? *Do you have any challenges in managing your horse? * Any financial challenges? *Weather challenges i.e., mud control *Horse health reasons which affect your management decisions?
Have you made any changes to your horse’s management? *Would you like to make changes? *When did you make these changes and why? *Were there any consequences (good or bad) to these changes?
Is your horse experiencing any health or behavioural issues? (i.e., lameness issues, respiratory issues, aggressive behaviours towards people or other animals, stereotypic behaviours (adding definition of what I mean by this). *Why do you think that may be?
Are you happy with the way you are managing your horse? Or are you happy with the livery management? *Why are you not happy or happy? *What would you like to change? *How do you think a change would impact your horse?
Blue sky question – If you could manage your horse in any way you choose, ‘no holds barred’, financially or otherwise, what would that look like? *Why would you choose that type of management? *Are there reasons why you couldn’t provide your choice of management for your horse, i.e. financial, current livery management, lack of availability?
Thank you for your time and do you have any questions for me or anything else you would like to say before we wrap up our conversation?

### Data analysis

The interviews were analysed according to Braun and Clarke’s (2019, 2022) RTA which is a qualitative research method developed by psychologists Braun and Clarke (2006) typically utilised to determine, analyse and interpret similar patterns (themes) within the observational data (in our study semi-structured interviews). RTA fully accepts the research’s subjectivity as non-biased and central to interpretation of the data.

WW coded the data and engaged in an iterative process for the identification and coding of the themes. The second author, (LJ) was not involved in the coding process. Both authors engaged in reflexivity throughout the analysis and reflected on their own subjectivities and world views. The first author as a previous livery and horse owner for many years and LJ, who was involved in the review of the codes and themes, is a social scientist and horse owner. Both LJ and WW were critical reviewers in the analysis and writing up stages and created an in-depth investigation of the different interpretations of the data between them as described by Braun and Clarke (2024). Both authors acknowledged that their experiences, beliefs and interests directly influenced their interpretation and decision-making, which shaped the current research. WW has owned horses for over thirty years, is a part-time PhD student with a Masters degree in equine science and was an Equestrian Canada riding instructor and a former owner, manager of two successful horse boarding facilities in Canada. She acknowledges that her equine experience and knowledge, particularly being a previous owner/manager of two horse boarding facilities, will have influenced the way she conversed with the participants and how she interpreted their responses and identified patterns/themes in the data. LJ is a psychologist in an animal welfare department, focusing on human behaviour change and human-animal interaction. LJ has also owned horses for over 30 years. In her review and interpretation of the themes LJ therefore reflected on how her experience of equestrian culture likely influenced her interpretation of owners’ experiences and beliefs. Both WW and LJ’s insider positions allowed for greater insight into the horse owners’ accounts, specifically the language that they used, the practices and the norms that they shared. This familiarity with the equine world/culture also carried a risk of over-identifying with the participants’ perspectives. To manage this, both WW and LJ kept reflexive logs, returning iteratively to the data to check interpretations against what the participants’ said.

The data were analysed through a social constructivist lens (Sivan 1986). Social constructivism recognises that individuals are active participants in the construction of their own knowledge and that knowledge is also gained through experience and social interactions (Andrews 2012). This means that in our analysis and interpretation of the data we acknowledged the importance of a participant’s experience and reality, as well as recognising that meanings, events and experiences are also the result of the different viewpoints that exist within a society (Andrews 2012). Discourse is an outcome of social interaction and is also the medium of exchange of social interaction. For example, what a person knows about their surroundings is linked to their own personal experiences and this is communicated through the interaction of language with others (von Glasersfeld 1989).

The data were coded and analysed in NVivo 11 by WW, using the six non-linear phases of thematic analysis: firstly, by becoming familiar with the data, then developing initial codes, searching for themes, evaluating those themes, followed by naming and defining the themes, and finally writing up the findings (Braun & Clarke 2019). WW first adopted a deductive coding approach whereby the data were coded according to the research questions, i.e. how do owners manage their horses, do they allow for the 3Fs and what are the reasons for their management approaches? In the second round of coding, WW also adopted an inductive approach looking for points of interest that were not necessarily related to, or driven by, our research questions, allowing for the coding of unexpected patterns and themes. Initial themes were then reviewed and discussed by both the researchers (WW and LJ). This process involved several rounds of discussions which resulted in further recoding, merging of themes and the changing of theme names. In line with the epistemology of RTA, we did not calculate an inter-reliability statistic because RTA is an interpretative approach that embraces the prospect of subjectivity in its analysis of the data. RTA does not claim generalisability of findings and therefore does not assume replicability of coding across researchers (Braun & Clarke 2019). Overall, the RTA identified the shared and nuanced experiences and challenges horse owners face in managing the 3Fs.

## Results

### Demographics

Most of the participants were female (n = 12) vs male (n = 2), were in the age ranges of 25–64 years (n = 14) and primarily located in England (n = 9), with (n = 2) in Ireland and (n = 1) each from Scotland, Wales and Northern Ireland. The majority of horse owners kept their horse at livery (n = 10) vs at home (n = 4).

### Themes

A total of five themes were constructed from the data. The themes were as follows: (1) ‘Choice for the horse is paramount’, (2) ‘Friends, forage, freedom and fear of injury’, (3) ‘When livery yard rules compromise horse welfare’, (sub-themes ‘Control of forage – access and quality’ and ‘Control of freedom – freedom of movement), (4) ‘Horse management through the (anthropomorphic) human lens’ and (5) ‘The livery yard – balancing both horse and human needs’.

#### Theme 1: ‘Choice for the horse is paramount’

Participants, who kept their horses either at a livery facility or at home, appeared to have a good understanding of the horse’s needs in terms of providing opportunity for their behavioural needs. When asked the ‘blue-sky’ question of managing their horses in whatever way they wished, most of the participants who kept their horse at livery referred to a track system method (Jackson 2007) as their optimal choice. Participants who kept their horses on their own properties largely described providing a ‘choice’ for their horses being of primary importance, e.g. providing access to shelter and free movement outside the stable environment was their ideal. The blue-sky question allowed the participants to express their “good horse owner” identities, whereby they placed choices for their horse as a priority, suggesting the horse needed to have the freedom to move and to forage with friends. For some, they felt having a track system would offer their horse all of these opportunities, as illustrated by the following quotes:

“*Yah, I would have the track system. And I would just feed him a high-quality hay analysed, supplemented* [for] *whatever is not in there. And yeah, I mean, he’s barefoot anyway. So, he would be barefoot on the sort of track system, which I would make a harder surface. He could socialise; he could move. It would just be a lot more natural* [being on a track system]” [Participant 5 L].

“*Oh, probably set up some kind of track system. Um so I think that kind of system where you know you maybe have access to grass that you can open and close, but where they’re kind of moving around to like, hay stations over like a variety of surfaces. If it’s really done well you sort of have different footing on different bits of your track. It’s a little bit, you know, how they would naturally live rather than just in a big square, grassy field. I mean, nothing is that natural but I think having to wander around a bit to look for food and going over different kinds of ground emulates nature a little bit more* [in a track system]” [Participant 7 L].

Conversely, the following participant felt that a track system did not provide enough choice for horses because they had tried the system previously and felt it did not provide enough freedom of movement for their horses:

“*I wouldn’t change. I wouldn’t. I can say I’ve tried a track system. But my experience of track systems is that they* [the horses] *don’t really move as much as I want them to move. There are reasons I like the idea of them, but in my opinion, they don’t move enough, even on a track system*” [Participant 12 H].

Another shared belief was that to ensure the horses’ needs are met, they should have choices in their environment. The following extracts illustrate the belief of choice in terms of access to shelter, shade and free movement when turned out. Most of these comments are derived from participants who kept their horses at home, which is perhaps why they had more opportunity to provide their horse with choices in their environment:

“*I suppose I feel like you know, these are animals that we have been domesticated, and they give an awful lot to us, and they do stuff that is unnatural. You know, I don’t kid myself that my horse would choose to be ridden every day or whatever. And for me, I think it is better for their health and their happiness if they can make decisions about where they want to be, what they want to do, where they want to graze. My horses seem very relaxed and happy. And I think it’s just better for their well-being altogether if they can make choices when they want* [to]” [Participant 10 H].

“*It’s just wrong, just totally wrong* [not to consider the horse’s needs]*. I just like them to be able to choose where they want to be. They can move around and choose where they want to be, whether they need shade or shelter. I like them to live out as much as possible*” [Participant 3 H].

#### Theme 2: ‘Friends, forage, freedom and fear of injury’

Some participants expressed a moral dilemma in terms of providing natural behaviour opportunities and safeguarding against injury. Others were specifically concerned when the livery yard did not seem to consider the importance of gradually introducing new horses to an established herd or if the livery management did not take extra precautions at feeding times to help prevent injury, as expressed in the following extracts:

“*Well*, [I am] *a bit anxious about moving the mare to the gelding’s yard just because she has to leave her pals and her current herd and be you know, be flung into a new herd. And I think that’s quite challenging in that the yard doesn’t have any introduction system. They just like wing a new horse in and right on with it. Yes. I mean, she* [my mare] *was already run through a fence* [at this livery]” [Participant 7 L].

“*That was a change* [in the management], *it was one of the reasons we left* [that livery]. *She* [the livery manager] *doubled the number of horses in the space for turn-out and* [the horses] *ate all the grass, which is where you got more conflict behaviour, more irritability. And yeah, if there’s big enough space or there’s enough food, you don’t normally…. they can get away* [from each other] *or there’s less of an issue”* [Participant 6 L].

The following participant reported that their livery management did not permit turn-out with other horses, preferring to keep the horses segregated in individual paddocks. The participant felt that this decision was possibly driven by concerns for injury. This participant also appeared to accept this decision, as long as their horse could still see other horses:

“*They don’t want it* [the livery]. *They* [the livery] *don’t want the horses to hurt each other. Yeah. I mean, they certainly have small electric fence between them so they can touch each other. But they’re not in the same field. But he’s always… he’s never alone, completely alone, that he can’t see any other horses*” [Participant 5 L].

#### Theme 3: ‘When livery yard rules compromise horse welfare’

The central organising concept of this theme was that the owners felt that their ability to maintain better equine welfare standards was limited by the control their livery yard exerted over husbandry decisions. The participants believed that their livery yard management played a significant role in whether their horse had access to the right type and quality of forage and turn-out. There was a clear sense of owner frustration, anger and/or concern around their lack of decision-making capacity. As these frustrations mostly extended to two of the welfare outcomes (i.e. forage and freedom), we abstracted this theme to two sub-themes to illustrate the context in which these were observed, i.e. 3.1 ‘Control of forage’ and 3.2 ‘Control of freedom (freedom of movement)’.

#### Subtheme 3.1: Control of forage – access and quality

There were shared participant beliefs regarding their horse’s access to quality forage, and access to appropriate amounts of forage. The following extracts illustrate some of these concerns:

“*My horse is on a small field, and he hasn’t had any grass for two to three months now. I mean, when we had snow for a week, I think they were not given any hay* [outside] *at all. She’s* [the livery owner] *aware of that* [the horse not having any hay] *and obviously, especially as I have asked her to give him hay because of that* [no grass when there is snow]” [Participant 5 L].

“*I mean, there have been occasions when they* [the livery] *fed stuff – six bales of absolute trash. And I’ve had to go and say, you know, please get that out my horse’s field. Because* [the livery manager] *hadn’t noticed basically the contractors* [the hay vendor], *that there’s six bales nobody looked to see what*…[quality it was]” [Participant 13 L].

“*I recently changed to hay because of variability in the haylage* [provided by the livery yard], *which is made on site and can’t be changed. It annoys me that the yard makes their own haylage, and bales vary greatly in their quality and acidity, which can easily be smelt. My horse can’t cope with the constant variation, gets a sore hind gut, and I have had to ask for him to be fed on hay instead*” [Participant 2 L].

There was also a belief that some livery yard management failed to see why too much grass for horses was a concern. Others expressed concern around controlling the amounts and types of forage their horses had access to, which are illustrated in the following extracts:

“*So, you know, she’s* [the livery owner] *quite into kind of preserving the grass as you would for sheep or cows. Not really ideal because over the summer, there’s a lot of issues with overweight horses at the yard. I think as a non-horse person* [the livery owner] *doesn’t recognise that most of our native types really need to be turned out onto like rough moorland. And you don’t want tonnes and tonnes of grass as you would for sheep or cows. …And yeah, the turn-out situation at the gelding’s yard um because of the way it* [the pasture] *is managed, and you’re just having too much grass to contend with. Not recognising that, that is actually problematic for a lot of these horses*” [Participant 7 L].

“*Yes, I do* [have a concern about laminitis] *And he’s* [my horse] *not really used to rich grass. They’re* [Andalusian breed type] *are not really bred to tolerate rich grass. But I am very aware that he shouldn’t be on too much grass or too much rich grass. I think they* [the livery] *fertilise the summer grass*” [Participant 5 L].

#### Sub-theme 3.2: Control of freedom (freedom of movement)

Under the overarching theme of livery yard rules, the control of freedom subtheme was observed, specifically regarding limited turn-out time for the horses. There was a belief that restriction of turn-out appeared to result from a need to preserve the fields for mud control or possibly a concern for overgrazing. There was also the perception that livery yards often simply failed to offer some level of flexibility in terms of turn-out options or that a lack of turn-out was due to livery rules and practices. These beliefs are illustrated in some of the following extracts:

“*Let’s see, from November until end of March, he’ll be in a stable, I’d say for about 21 hours a day. Yeah, so there would be no alternative field, so there’s nowhere that they could rotate. So, there’s no turn-out really, in the winter it is hard standing for a couple of hours. And that’s it. It* [the field] *would get poached, you would ruin the field by summer. It’s very difficult not to, I think in the climate that we have, not to get it* [the field] *a bit ruined, but some people* [other liveries] *actually use a little bit of a system where in which they use one field and then they’ll move on to another and give the other field a time to recover. But they don’t do that at this livery*” [Participant 11 L].

“*Oh, that’s the yard rules they can either be out overnight or out in the day but they can’t be out 24/7. If you ask* [the livery owners], *they will say it’s about saving the fields*” [Participant 7 L].

#### Theme 4: ‘Horse management through the (anthropomorphic) human lens’

This theme was constructed from the participants’ discourse that appeared to reflect anthropocentric and anthropomorphic beliefs. Some owners felt that their needs/wants should outweigh the needs of their horse (anthropocentric beliefs) even when the owners appeared to understand the behavioural needs of the horse. These attitudes appeared to result from the participants’ motivational needs, such as keeping the horse in overnight to avoid them getting dirty, the wish to make use of the livery facilities and managing their horse, i.e. lack of freedom of movement, in a particular way for their own convenience:

“*He was in at night to suit me. I don’t like cleaning wet mud off horses before I ride them, or trekking 200 m down a field to fetch a horse in on a cold, wet winter morning*” [Participant 2 L].

“*I still think I would always want to keep him in at night. Because he’s a sports horse, and you kind of lose some of that fizz if they’re out 24/7. So, he’s always moving, which is great for his joints, but I think, there’s a noticeable difference when he’s been stabled as to when he’s been out for a day or a few days, or if you’re wanting them to have that kind of hyped-up edge ready to go in to jump. He’s getting to kind of chill* [in the stable] *to conserve his energy. So, when I do take him out, he’s kind of ready to go*” [Participant 11 L].

Anthropomorphic beliefs also appeared to underpin people’s management decisions with regard to freedom of movement. For example, some participants felt that their horses would “not like” to be turned out in wet weather or to stand on slippery footing or in mud. One participant felt that a tolerance of unpleasant weather was also breed-specific:

“*And most of the horses don’t really wanna be out all day, if it’s horrible weather. So, there’s a hope he may stay out longer though, because I know that would suit him* [to be outside] *better in the middle of winter. He would like to go out and run around. But if it’s raining, he won’t want to go out. It’s… yeah, if it’s nice weather, he will happily turn out and stay out. But I think if it’s really wet and yucky, it’s the ground, the ground. I don’t think he appreciates standing on wet ground. He’s not like, yeah, a Welsh cob who’s more used to it. I don’t think he likes it. He doesn’t like slippy ground and I don’t think he likes standing in mud*” [Participant 6 L].

“*During the summer, he’ll get turned out I would say most days. Unless the weather’s terrible, because I don’t want him getting wet*” [Participant 11 L].

The following participants also assigned human behaviour traits to their horse, likening equine behaviour to human behaviour, e.g. labelling their horses as ‘greedy’, which may have affected the horse’s access to a forage source. Another participant felt that their horse displayed dominance in social interactions with other horses and used negative human traits to describe them:

“*I mean some horses. Not all horses are greedy, but mine are greedy. Although I have been criticised for calling horses greedy - but mine are greedy. But they are Spanish horses they come from a place which does not have grass at all most of the time, you know, it’s basically a desert type area. They have to be watched for this* [over eating grass] *and the grazing has to be limited because of that. I’ve had Connemara* [ponies] *here, which were little piggies. I’ve the cob that would be the size of my house if I let him stuff his face, you know?*” [Participant 12 H].

“*But my Irish sports horse, is so mean to her* [the owner’s other mare]. *She’s just like…you know ‘you’re my bitch and I could do what I like with you’. It’s this horrible, this co-dependency type thing. She particularly hates chestnut horses, and she would chase them off. She hates them. She’s a very, very, very opinionated horse*” [Participant 13 L].

#### Theme 5 – ‘The livery yard – balancing both horse and human needs’

Participants expressed mixed feelings about their livery yard management. Some participants appreciated the facilities offered by their yard, whilst others voiced their unhappiness with their current livery management. Two participants were very concerned about the welfare of other horses kept at the same livery yard, because these horses did not receive consistent care or visits from their owners:

“*And the owner very seldom visits, and the horse stands in the rest of the time. I think this year*, [this horse] stands in almost probably 23 hours out of every 24. And they’re standing in sh*t most of the time. And they get fed in the mornings, and they get their forage in the mornings. And then by the time I get up at lunchtime, they [the horses at the livery] *have nothing to eat. So, they’re standing there with no feed, nothing to eat until maybe six o’clock in the evening*” [Participant 9 L].

“*There are horses there* [at the livery] *whose owners never come, it’s like a retirement, and I have in the past, had to call the* [well-known horse charity]. *The welfare officer came and had a chat* [with the livery owners]. *You know, so obviously, I did that anonymously. Um, so yes, there are some occasions when there has not been enough food at all and I’ve had to give my horse supplemental feeding because they* [the livery] *just won’t give them enough. Yeah, I know, it’s horrible, isn’t it?*” [Participant 13 L].

Another participant discussed how they are mostly happy with the way their horse is kept at livery, as well as the livery facilities, but they also expressed being much less satisfied with their own emotional and social needs:

“*No, not really. No, I wouldn’t say for the most part. I am quite unhappy. There are advantages. You know, apart from the hay, he’s well looked after. And the facilities are great. The welfare is good. Apart from the hay, part. The hacking is there. There’s contact with the other horses. But, yeah, apart from that, no, I’m not I’m not happy really, at all. No. It’s not friendly at all. Yeah. And that’s what I thought it would be. I thought it would be friendly, but they are…the owner, and one of the liveries are very close, then the other livery person is never there. But I have got nobody and nobody wants to do anything with me. Yeah. It’s really not very nice*” [Participant 5 L].

There were participants who were happy with their yards and voiced fewer concerns around control and constraints. These participants, for example, described such things as having access to space for their horse, or that their horse appeared to be happy and exhibited calmer behaviour in their current livery yard:

“*He’s* [my horse] *very happy there. He’s very, very settled. The previous yard* [we] *moved from, which had some wonderful hacking, he wasn’t settled or wasn’t happy. He seems happy* [at the current livery]. *He’s calmer. There’s less unwanted behaviour*” [Participant 6 L].

“*They’ve got plenty of space. They’ve* [the horses] *got room to move around. It’s easier for me. This is actually on the yard, because my problem is mobility and going backwards and forwards, was hard for me. But, you know, I’ve got a lot of support* [from the livery]. *So, of all the yards that I could have found it turned out to be the best yard, and because I don’t feel safe riding anymore. I started him driving and they’ve been able to do that. So, it’s been a bit of a dream come true to be fair and only four miles from home and I can’t believe it*” [Participant 3 L].

## Discussion

Our study identified several key factors which prevented horse owners from providing their horses with the 3Fs. Nearly all of the participants, whether they kept their horses at livery or at home, did believe that providing a horse with the opportunity for choice in their environment was paramount for good welfare. Being able to engage in best practices appeared to be most difficult for those who kept their horses at livery yards. Livery yard constraints and the horse owner’s anthropocentric/anthropomorphic and motivational beliefs, all appeared to underpin horse management practices. Most participants in our study kept their horses at livery, which also played a significant role in the management of their horses and therefore also understanding livery yard management practices is an important consideration for improving a horse’s opportunity to the 3Fs. However, it is also important to note that we are reporting on the horse owners’ beliefs, attitudes and experiences and, as we did not conduct interviews with livery managers, and we do not therefore have the same perspectives as those from the livery yard management.

### Choice for the horse is paramount

Participants expressed the shared belief that their horses’ behavioural needs are important and believed that provision of choice in the horse’s environment was paramount. Some participants felt that a track system (Jackson 2007) would meet their horse’s behavioural needs. Others felt that being able to provide choice for their horse, such as allowing the horse to “*make decisions about where they want to be, what they want to do, where they want to graze*”, with access to shade or shelter, was a priority. A track system, also described as ‘paddock paradise’ is believed to meet the 3Fs because it is designed to promote movement, social interaction, access to forage, and aid in weight management of horses (Jackson 2007). Kirton *et al.* (2025) found that ponies moved more and displayed less antagonistic behaviour when managed on a track system vs a strip grazing system. A track system also compares favourably against traditional forms of horse management, which often consist of individual stabling and provide little social interaction or turn-out (Furtado *et al.* 2022; Cameron *et al.* 2025). However, it is not clear in all examples if the participants who wished to use a track system had any previous experience of using this type of management system. Currently, there is also very little research which thoroughly examines the pros and cons of a track management system. Although track systems may be an increasingly popular form of management for horses, not everyone we interviewed agreed they were beneficial, with one participant, who had previously utilised a track system, reporting that they felt a track system did not provide enough movement for their horses. This participant’s concern is consistent with findings by Farmer *et al.* (2023 who found that horses managed on a track system did not show any increased movement when measured with Global Positioning System (GPS), compared to those horses managed on conventional pasture. Further investigation is needed to understand the benefits of track management system and the benefits to horse welfare.

Other participants expressed a shared belief that providing choices for their horse with regards to opportunities for freedom of movement and access to grazing, was of paramount importance. The provision of autonomy and agency, i.e. providing a choice for animals has been identified as a good step towards meeting the animals’ welfare needs and promoting good health and good welfare (Stamp Dawkins 2021; Englund & Cronin 2023; Goodale 2026). Rault *et al.* (2020 defined positive welfare as “*the animal flourishing through the experience of predominantly positive mental states and the development of competence and resilience*”. Therefore, it could be argued that our current findings align well with the goals of positive welfare as our participants emphasised that the provision of choice for horses was a very important aspect of good welfare.

Other studies have suggested that lack of knowledge was a limiting factor in providing good welfare for horses (Goodwin 1999; Horseman *et al.* 2016; Rioja-Lang *et al.* 2020; Hemsworth *et al.* 2021; Mauricio *et al.* 2024; RSPCA Report 2025). These inconsistencies most likely stem from differences in research methods, participant samples, and how welfare and knowledge may have been operationally defined. However, even when horse owners do appear to know and understand what best practice care and management is for horses there also appears to be a gap between this knowledge and the implementation of best practice management (Visser & Wijke 2012; Ross *et al.* 2023; Mauricio *et al.* 2024). There is a paucity of research clearly identifying the ‘knowledge and follow through in practice gap’ and the reasons for this gap are likely to be multifaceted. A lack of financial resources as described in Mauricio *et al.* (2024) could be a contributing factor as well as a lack of motivation to change behaviour due to tradition or ingrained habits, i.e. “*performance of strong habits may not be dynamically responsive to current motives*” (Neal *et al.* 2011; Watney *et al.* 2025). Also, the lack of opportunity to follow through with needed change may interrupt the knowledge and follow through gap (Michie *et al.* 2014). For example, horse owners may possess the knowledge required to implement best practice management of the 3Fs but could be lacking the opportunities to implement change due to livery management decisions or practicalities, as some of our participants reported. These participants believed that their capacity to provide optimum care for their horses lay beyond their control and expressed concern about livery management decisions around turn-out with other horses, the quality and quantity of forage provided, and the general level of care provided for horses at their livery yard. It may therefore be the livery management who lacks optimum equine welfare knowledge, according to some of our participants’ experiences. The RSPCA (2025) report suggested that there was a lack of knowledge and expertise in livery establishments in England and Wales and therefore the provision of the 3Fs may have also been neglected for these reasons. However, as we interviewed the horse owners and not the livery yard management, a lack of livery yard knowledge was not investigated in this study. It could also be that the some of the livery’s management decisions may have stemmed from financial or infrastructure constraints, which is consistent with the findings of Mauricio *et al.* (2024).

### Friends, forage, freedom and fear of injury

Access to conspecifics in the current study was often linked to the provision of forage and turn-out. Our participants also expressed concerns for injury when the livery yards failed to provide a sufficient amount of forage for the number of horses present in the field during feeding time. Provision of sufficient amounts of forage for the number of horses present when turned out together may help prevent injury between horses. According to van Dierendonck (2006) there needs to be adequate space and foraging opportunities in turn-out for every horse in the group to encourage positive social interactions in a herd dynamic and prevent the risk of injury. Such opportunities may result in less aggressive behaviour and risk of injury between horses during provision of forage, but this may be a challenge for liveries with a large number of horses and restricted space for turn-out, which was noted in the RSPCA study (2025). There was also a shared concern that livery management prevented the participants’ horses from having access to other conspecifics, largely due to the concern for injury even without reference to the provision of forage. Previous research suggests that injury is not uncommon when horses have been turned out in groups (Knubben *et al.* 2008; Owen *et al.* 2012; Hartmann *et al.* 2015a) and therefore, the concern of the livery yard in restricting access to conspecifics, especially concerning liability, may not be surprising. Horse welfare research does strongly support turn-out with conspecifics (van Dierendonck 2006; Hartmann *et al.* 2012; Sigurjónsdóttir & Haraldsson 2019; Phelipon *et al.* 2024), but also the stability in groups during turn-out and the planning of the composition for the group are important considerations for preventing injury and promoting good welfare (Hockenhull & Creighton 2014; Sigurjónsdóttir & Haraldsson 2019).

One participant felt their horse’s welfare was not compromised if their horse could always see, but not necessarily touch, other horses, due to the livery yard preference for not turning horses out with conspecifics due to a concern for injury. Electric fencing is often deployed in horse facilities to prevent tactile contact between horses, but the negative consequences of the use of electric fencing on horse welfare has not been well researched (Glauser *et al.* 2015). Although there is a significant body of literature pointing to the welfare impacts of impeding a horse’s innate need for social interaction (van Dierendonck & Goodwin 2005; van Dierendonck 2006), knowing how these “see but not touch” husbandry scenarios impact the horse, requires further investigation.

### When livery yard rules compromise horse welfare

The participants expressed their shared frustrations at not being able to provide their horses with the right type, quality or access to adequate amounts of forage or adequate turn-out time for their horses.

### Access and quality of forage

Some of the participants perceived that livery management control around the provision of forage often involved restricted access to a forage source, concerns over forage quality, or the variability of the forage. This is a legitimate concern as lack of access to a fibrous forage source for extended periods can result in ulcers and/or colic in the horse (Scantlebury *et al.* 2011 Ermers *et al.* 2023). The variation and quality of the forage source provided by the livery management was also a source of frustration for participants as some felt their horses’ health may have been impacted due to the changing quality of haylage; whilst others were disappointed by the consistency and quality of hay their horse received. Variability in forage, such as inconsistent sources of hay or haylage (Muller *et al.* 2011), and poor-quality forage sources, i.e. dusty or mouldy hay (Intemann *et al.* 2022), have been found to impact the horses’ respiratory and digestive systems. Therefore, this research suggests our participants concerns are justifiable.

The type of rich pasture provided by the livery and the management practice of ‘preserving the grass’ as is done for production farm animals was also a concern. There is good reason for the participants’ apprehension regarding this matter since horses exposed to rich pasture can be at risk for obesity, laminitis and metabolic disease (Bott *et al.* 2013; Wylie *et al.* 2013). Past research has shown that horse owners in general lack knowledge of the type of forage and fibre requirements needed for optimal horse health and welfare (Ermers *et al.* 2023). Therefore, it is possible that a lack of knowledge on the part of the livery management with regard to provision of the right type of forage sources for horses, may also provide some explanation for their practices in the current study. Lack of communication around the provision of forage between livery management and the participants also appeared to create a sense of mistrust that permeated the discourse between horse owner and management. As a negative feedback loop exists between mistrust and poor communication (Savolainen *et al.* 2014), this issue of perceived mistrust could impact the welfare of the horse. Others have also found that effective communication between the livery yard management and horse owners was a key factor to ensuring good welfare for the horse (Birke *et al.* 2010).

### Restriction of freedom of movement

A lack of space and field conditions appeared to play a pivotal role in restriction of turn-out by the livery yards. This finding is also consistent with the reporting of others regarding a lack of field space for turn-out and too many horses (Birke *et al* 2010; Boden *et al.* 2013; Longland *et al.* 2013; RSPCA 2025). Participants also reported that preservation of the fields due to wet and muddy conditions was a common reason for their livery yards not accommodating turn-out in the winter, which was also cited in other studies (Birke *et al.* 2010; Furtado *et al.* 2021).

### Horse management through the (anthropomorphic) human lens

The human lens theme revealed that both anthropocentric, anthropomorphic and motivational beliefs appeared to influence restrictions of the 3Fs. Anthropocentric beliefs suggest that human experience is the principal reality and that human behaviour should take precedent over animal needs and behaviours (APA 2018). Anthropomorphism can be described as an individual attributing human characteristics to non-human animals and objects (Mota-Rojas *et al.* 2021). Anthropomorphic beliefs have been commonly reported in horse-human interactions and management, and such beliefs can become a concern when they impact upon the horse’s welfare needs (Birke *et al.* 2010, Hausberger *et al.* 2021; McKee 2023; Fielder *et al.* 2024; Merkies *et al.* 2024).

One participant’s anthropomorphic language included describing their Cobs and Connemara ponies as being “greedy”, and “little piggies”, “stuffing their faces” and “becoming as big as houses” in the context of access to grazing. Borkfelt (2011) proposes that labelling non-human animals, with both generic and specific names, can negatively impact our relationships with them and Goatly (2006) suggests the offensiveness of animal metaphors promotes the ideology of human superiority over animals. Therefore, how we refer to non-human animals demands careful consideration. Based on this participant’s comments, it is likely that these horses’/ponies’ access to grazing was limited to control weight. This decision could be warranted because some native horses and ponies have been found to be at greater risk for developing equine metabolic disease (Furtado *et al*. 2022) which could be a reason for some owners to restrict access to unlimited grazing. However, there is also a caution for welfare because movement and grazing are behavioural needs in horses and ponies and if either is strictly curtailed, the impact could lead to the expression of rebound and frustrated behaviours (Hughes & Duncan 1988). In another extract, a participant described their horse as “bitchy”, “dominant and opinionated” and “hated chestnut horses”. Such types of negative anthropomorphic beliefs, associated with mares specifically, have been found to lead to more severe forms of training methods based on the horse’s sex (Aune *et al.* 2020). Even in general terms, horse owners often describe behaviour in female horses as ‘mareish’, which often translates to horses being described as recalcitrant difficult, moody and flighty (Curry *et al.* 2007; Dashper *et al.* 2018).

Another shared anthropomorphic belief revolved around participants’ feelings regarding wet weather or slippery footing and holding the opinion that their horse “would not want to go out” or that “he [the horse] wouldn’t like it”, or “I don’t want him getting wet”; even though one participant acknowledged that an increase in turn-out would be in the horse’s best interest. Projecting our own negative beliefs about the weather, such as a dislike for rain, could therefore have a negative impact on the horse’s opportunity for freedom of movement. People may not wish to stand out in the rain, but is that the same choice a horse would make? One study found that horses preferred to wear a rug in wet weather (Mejdell *et al*. 2019) whilst another study identified that when the speed of the wind was > 2.8 m s^–1^ and accompanied with rain, there was a greater likelihood of horses seeking shelter (Heleski & Murtazashvili 2010; Snoeks *et al.* 2015). However, when provided with a choice of outside access, horses spent on average less than 3 h, non-consecutively, in a 24-h period inside their stable (Lansade *et al.* 2025). During the summer, this amount of time doubled which suggests that horses may prefer to spend more time inside, in a shaded environment, when it is warmer or to avoid insects (Heleski & Murtazashvili 2010). Other studies have also shown that horses will utilise shelter, not only when turned out in wind and rain, but also in the summer sun (Hartmann *et al.* 2015b). Therefore, provision of the choice of shelter during turn-out could provide reassurance for horse owners about their horse’s comfort and may increase the chances of the horses receiving more freedom of movement, despite the weather conditions. However, it is also conceivable that some horse owners may wish to keep horses in during wet weather to prevent slips, injuries and health issues for both horses and people.

One male participant curtailed their horse’s freedom of movement because they felt that their horse “jumped better” after being kept in the stable for extended periods of time, “giving the horse a hyped-up edge, ready to go in to jump”. This participant opted to keep their horse in the stable, if given the option, and was therefore preventing his horse’s access to freedom of movement in preference of increasing a perceived competitive edge. Werhahn *et al.* (2011) suggests this is not an uncommon theory amongst equestrians but also found that a horse’s willingness to perform is not affected by their access to free movement. Werhahn *et al.* (2011) also noted that are many determents to confining a horse to its stable, such as abnormal behaviours, and diseases of the musculoskeletal and respiratory systems.

Interestingly, Jones (1983) described horse owners who were more interested in horses from a riding and sports perspective as “achievers” and those who were more interested in achieving a personal relationship with their horses as “relators”. Jones also found males were more likely to connect to their horses through sport and competition. Similarly, Luke *et al.* (2024) found that equestrians were largely goal-oriented and were highly motivated to compete or participate in club activities with their horses. Therefore, the culture of competition, which is associated with equestrianism, may influence the decisions around husbandry, with some owners paying less attention to the behavioural needs of their horse in order to accommodate their own motivational needs as a competitor/rider.

Under the theme of the livery yard balancing both horse and human needs, one of our participants reported feeling socially isolated at their livery yard. The participant seemed to accept this situation because most of the care their horse received at their livery yard appeared to be more important to them. Livery yard management practices have been previously cited as having negative impacts on both equine and human well-being (Birke *et al.* 2010; Smart 2014) and, although only one participant specifically reported feeling socially isolated, other participants also reported negative emotions associated with their livery yards. Livery yards are organisations in which the different opinions and habits of horse owners, proprietors and staff meld together to create a social structure and influence (Smart 2014). Livery yards are akin to an organisational culture which can be described as the beliefs and expectations collectively held by an organisation’s members (Chalmers *et al.* 2025). Furtado *et al.* (2021) found that management strategies and horse care was extremely variable between different livery yards, and that ‘livery cultures’ were a product of the livery owner’s own beliefs of what constituted good horse care. Furtado (2023) also suggested the culture of the livery yard is influenced by yard owners’ and managers’ preferences, such as keeping horses in stables as a means of comfort and safety, and restriction of conspecifics in turn-out which was also a factor in our participants’ experience of livery management. Will (2016) suggests that horse owners keep their horses at livery to enjoy and care for their horses as well as participate in similar activities, but also that livery yards can often be highly competitive, status-driven and sometimes unpleasant places to be. Sometimes relationships between the yard owners, yard staff, the clients, and the horses can result in negativity, which in turn impacts both the human and horse welfare (Will 2016). Therefore, it is possible that livery yard culture may have impacted horse husbandry decisions which affected our participants’ experiences. However, it should also be noted the participants’ opinions are one-sided and other studies have shown that horse enthusiasts are sometimes keen to ‘point fingers at others’ and place emphasis on what they think others are doing wrong (Luke *et al*. 2023; Copelin & Merkies 2025).

Two participants felt that the livery management were neglecting horses in their care because their owners rarely visited them and therefore could not or did not oversee their horse’s well-being. It was a shared belief that these ‘forgotten’ horses were not receiving enough forage or turn-out and were being confined in dirty stables for long periods of time with no food, so much so that the welfare issues for one of these horses was anonymously reported to a well-known horse charity. Reporting this type of incident may have provided some relief and reassurance for the participant, but it is unknown if a permanent change in management for the horse resulted from this consultation.

However, not all participants reported negative experiences at the livery yards. Comments from participants about support of their mobility issues from their livery yard, and the closeness in vicinity to their own property ‘being a dream come true’. Other positive responses noted included horses being happier at a current livery yard, as opposed to a previous one.

In summary, there were several contributing factors which prevented our participants’ horses being provided with the 3Fs. Whilst many participants believed that a choice for their horses was paramount, they may have been prevented from doing so because of their livery management’s practices or rules. The lack of access to conspecifics appeared to be centred around a concern for injury and liability, not only from the livery yard’s perspective, but also the participants’ concerns. Possible reasons for horses not receiving the right type or quality of forage could be the result of the livery yard’s lack of knowledge or motivation to provide the horse’s optimum forage requirements. Participants’ anthropocentric, anthropomorphic and motivational beliefs also appeared to impact the horse’s access to the 3Fs.

### Study limitations

Qualitative research is often criticised for researcher subjectivity and bias, and difficulty in reproducing general applicability (Mwita 2022). Reflexive Thematic Analysis (Braun & Clarke 2019) however, recognises the researcher’s introspective and reflective approach to their data and the analytic process. Subjectivity is therefore recognised and embraced as part of the analytical process. RTA does not value objectivity commensurate with the dataset, but rather trusts in the subjective, immersed engagement of the researcher with their data, in addition to their own experiences and perceptions (Braun & Clarke 2021a,b).

Our sampling approach involved using online social media to recruit the participants for the interviews and this approach will not have included those without technological ability or online access and therefore these individuals would not be able to take part in the research. Those individuals with a greater interest in welfare for their horses may have been more likely to respond to our request for interviews which suggests that participation bias and/or self-selection bias (which proposes there are very different characteristics between those who volunteer for studies to those who do not; Alarie & Lupien 2021), may have played a part in influencing our outcomes.

Most of the interviews were carried out online via Microsoft Teams® and recorded at the time of the interview. However, there were two participants who did not wish to be recorded and therefore one interview was carried out via email over the course of a few days and the other conducted via WW’s iPhone with notes taken during the interview and then subsequently transcribed. It was important to take the participants’ preference for the interviews into account, but these two interviews were demonstrably different to the recorded Teams/Skype® interviews. Regarding the note-taking, either from the email response or taking notes whilst listening to a participant speak, it is possible that the effectiveness of the communication may have been disrupted between the interviewer and participant due to the tone of voice being lost (Muswazi & Nhamo 2013).

We completed 14 interviews, which we considered sufficient to provide rich and valuable data. Data saturation is also often considered as a limitation in qualitative research. However, Braun and Clarke (2021c) also suggest that there is often a level of uncertainty around achieving a definitive point of saturation in qualitative research and suggest that decisions regarding sample size and when to complete data collection, are subjective and influenced by the researcher’s interpretation of the data and their own judgement. These authors also suggest that data saturation, which is traditionally discussed in qualitative research, is not consistent with the assumptions and values of RTA. In essence, saturation predicts a set of meanings that are fixed, finite and subject to exhaustion, which is not compatible with the Big Q approach of RTA (Braun & Clarke 2024). It should be noted that the results of this study are not intended to be generalisable but to simply play a part in gaining further understanding of the variety of welfare challenges faced by certain horse owners in the UK and Ireland.

### Animal welfare implications

Horse owners in our study reported that providing choice for their horses is paramount for good horse welfare. These results suggest that our participants had a decent understanding of their horses’ innate welfare needs, which is an encouraging finding. However, previous research has also shown that even when horse owners understand and are aware of their horse’s welfare needs, such knowledge is not always acted upon in practice. Anthropocentric, anthropomorphic, motivational beliefs, livery culture and management practices may have hampered access to the 3Fs in our study. Further research to ascertain livery management challenges with regard to the behavioural needs of horses and the provision of the 3Fs would be beneficial in understanding the gaps in horse welfare. An effective human behaviour change intervention, such as training livery managers and horse owners on safe ways their horses can be turned out with conspecifics, for as long as possible, and education on the provision of the right type and amount of forage for horses, may be successfully employed if the opportunities and motivations behind the horse owners’ and managers’ actions are understood and considered.

## Conclusion

Overall, it appeared that many of our participants had a good understanding of the horse’s behavioural needs and voiced a preference for track systems and choices for horses, suggesting that the horse’s welfare needs were important to them. Our research has also been instrumental in identifying some of the key factors which hamper the opportunities to provide the 3Fs for horses. Livery yard control, and individual factors, such as the anthropocentric/anthropomorphic and motivational beliefs, all appeared to create challenges in providing opportunities for the 3Fs. The livery practices surrounding turn-out and conspecifics, lack of turn-out opportunities and the quality and inadequate access to forage source, all caused frustration among participants. Our evidence suggests that some of the reasons for these shortfalls could have been due to the lack of knowledge on the part of the livery management.

Satisfaction with the livery yard varied across participants. Some reported being very happy with the facilities and the care provided for their horses. One reported overall satisfaction with their horse’s livery provision despite they themselves noting a sense of social isolation. However, our results were self-reported from participants about livery yard experiences, and it would also have been beneficial to have had more information on management challenges from the perspective of the livery owners themselves in order to have gained a better understanding and overall picture of all the challenges associated with meeting the 3Fs for the horses. There is currently very little research focusing on the challenges faced by livery establishments and the reasons behind their management choices. To improve welfare for horses based on their behavioural needs, further research examining how human behaviours can be changed to positively influence management and horse welfare is required. This could be a human behaviour change strategy, which targets the specific aspects and behaviours, such as environmental context and resources or belief about capabilities, which are preventing livery and horse owners from providing their horses with more access to freedom of movement, and access to conspecifics.

## Supporting information

10.1017/awf.2026.10107.sm001 Watson et al. supplementary material Watson et al. supplementary material

## Supplementary material

The supplementary material for this article can be found at http://doi.org/10.1017/awf.2026.10107.

## Long descriptions

### Table 1. Long description

The table consists of a single column titled Interview schedule containing ten numbered sections.

1. Ice breaking conversation including a welcome and a question about the weather.

2. Ownership details: Number of horses, age, breed, sex, duration of ownership, and whether multiple horses are managed similarly or differently.

3. Activities: Types of competition (dressage, show jumping), leisure, hacking, and how these activities affect management.

4. Management specifics: Control of management, turnout frequency and duration, social contact with other horses, forage types (grass, hay, haylage), and barriers to providing more friends, forage, and freedom.

5. Challenges: Financial, weather (mud control), and horse health reasons affecting decisions.

6. Management changes: Past or desired changes, timing, reasons, and consequences.

7. Health and behavior: Issues such as lameness, respiratory problems, aggression, or stereotypic behaviors.

8. Satisfaction: Happiness with current management or livery and the perceived impact of potential changes.

9. Blue sky question: Ideal management scenario without financial or practical constraints and current barriers to achieving it.

10. Conclusion: Closing thanks and an invitation for participant questions.

Navigate back to Table 1..
