# Supplementary material for: Choice for the horse is paramount: Owners’ experiences in facilitating friends, forage, and freedom in their management practices
Source: Anim Welf. 2026 Aug 10;35:e48. doi: 10.1017/awf.2026.10107 (PMC13462703; doi:10.1017/awf.2026.10107)
Supplement: Supplementary file 1 [file S0962728626101079sup001.pdf]

THE UNIVERSITY of EDINBURGH
The Royal (Dick) School
of Veterinary Studies

The Jeanne Marchig
International Centre for
Animal Welfare Education

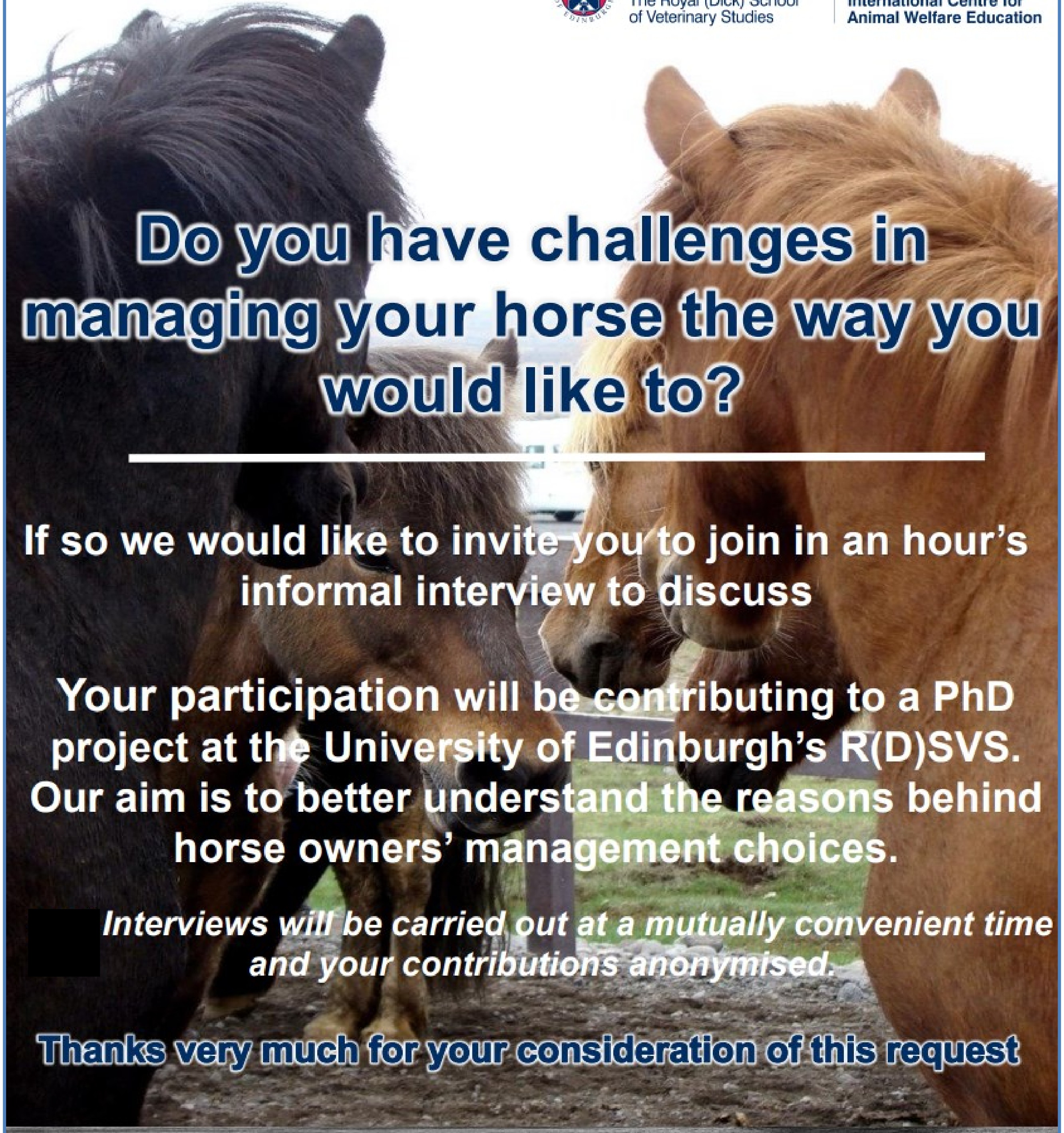

Do you have challenges in managing your horse the way you would like to?

If so we would like to invite you to join in an hour's informal interview to discuss

Your participation will be contributing to a PhD project at the University of Edinburgh's R(D)SVS. Our aim is to better understand the reasons behind horse owners' management choices.

[redacted] ***Interviews will be carried out at a mutually convenient time and your contributions anonymised.***

Thanks very much for your consideration of this request

To participate you must be 18 years or older, a resident of the UK or Ireland and own or manage at least one horse

Click here to find out more

Challenges to Horse Management - PhD Interviews

Page 1: Privacy Notice

THE UNIVERSITY *of* EDINBURGH
The Royal (Dick) School
of Veterinary Studies

The Jeanne Marchig International Centre for Animal Welfare Education

Hello and welcome to this information sheet regarding a request to participate in a PhD interview on the challenges to horse management.

These interviews are part of a PhD research project investigating the current health and management of horses and ponies in the U.K. and Ireland.

The data collected here will be used to form part of the PhD, and some of the data may be used in other ethically approved research. Anonymised data may be reported at conferences or in research papers to help other stakeholders learn from our experience.

This data **will not** be used to identify you as an individual and will be managed according to GDPR (General Data Protection Regulation). The interview will take approximately 50- 60 mintues to complete and will be arranged at a mutually convenient time.

If you do not wish to take part, you can close this browser window now. If you would like to know more about how we will use your data, you can read the statement below.

Page 2: Invitation to Participate - PhD Interview - Challenges to Horse Management - Privacy Notice

Why are we running these interviews?

We want to know the challenges people in the U.K. and Ireland have with their horse management choices; in order to gain a more detailed insight into current horse keeping practices.

Who is responsible for these interviews?

Wendy Watson BSc.(Hons), MSc. part-time PhD student at the University of Edinburgh, is responsible for the interviews.

This survey is being managed by Wendy Watson email: UofEdEquinePhD@ed.ac.uk

Who will have access to my responses?

- Wendy Watson, who is the primary researcher, as well as the members of her research team as listed below.
- Professor Cathy Dwyer, Dr. Jill MacKay, Dr. Kirsty Hughes, Dr. Patrick J. Pollock, Royal (Dick) School of Veterinary Studies, University of Edinburgh

What if I decide I want to withdraw my response?

Participation in this project is voluntary, and there will be no financial benefit for participation. Participants can withdraw at any time by contacting UofEdEquinePhD@ed.ac.uk

There is no penalty for withdrawing and respondents will be informed of this prior to accepting the invitation to participate.

The project and associated data collection methods are not intended to make participants feel uncomfortable; however, if participants do feel uncomfortable, they are encouraged to make Wendy Watson, the PhD candidate, aware. In the first instance, Wendy can alter the questions and redact partial responses, but if the participant wishes, they may exit the project.

When will be data be destroyed?

The data will be destroyed 3 years post the end of the evaluation project (January 2027).

If you wish to continue, please tick the box on the next page, provide the answers to a few demographic questions and add your email address when prompted for contact. Many thanks indeed for your time.

Please tick to confirm you agree to participate in the interview (to be scheduled at a mutually convenient date/time)

- ○ I agree

I am over 18 years of age.

- ☐ Yes

I define myself as being:

- ☐ Male
- ☐ Female
- ☐ Prefer not to say
- ☐ In another way

Are you a resident of the U.K. or Ireland? *Required*

Please select exactly 1 answer(s).

- [ ] Yes
- [ ] No

Which location in the U.K. do you live in?

- [ ] England
- [ ] Scotland
- [ ] Wales
- [ ] Ireland
- [ ] Northern Ireland

Do you own or manage a horse(s) or pony?

- [ ] Yes
- [ ] No

Do you keep your horse at home or livery (most of the time)?

- [ ] Home
- [ ] Livery

Contact Information:

Please enter a valid email address.

Page 3: Many Thanks for Your Help - We are Grateful for Your Support of This Research
